# Genetic investigation of the association between maternal dietary patterns and offspring ADHD

**DOI:** 10.1038/s41380-026-03645-w

**Published:** 2026-05-25

**Authors:** Kristina Aagaard, Casper-Emil T. Pedersen, David Horner, Anders Eliasen, Nicklas Brustad, Rebecca Vinding, Mohammad Talaei, Seif O. Shaheen, Julie B. Rosenberg, Jakob Stokholm, Bo Chawes, Morten Arendt Rasmussen, Jens Richardt M. Jepsen, Bjørn H. Ebdrup, Alexandra Havdahl, Laurie J. Hannigan, Klaus Bønnelykke

**Affiliations:** 1 COPSAC, Copenhagen Prospective Studies on Asthma in Childhood, Herlev and Gentofte Hospital, University of Copenhagen, Copenhagen, Denmark; 2 Division of Endocrinology, Boston Children’s Hospital, Boston, MA USA; 3 Medical and Population Genetics, Broad Institute of MIT and Harvard, Cambridge, MA USA; 4 Department of Pediatrics and Adolescent Medicine, Copenhagen University Hospital - Herlev and Gentofte, Herlev, Denmark; 5 Department of Clinical Medicine, Faculty of Health and Medical Sciences, University of Copenhagen, Copenhagen, Denmark; 6 Centre for Preventive Neurology, Wolfson Institute of Population Health, Queen Mary University of London, London, UK; 7 Allergy and Lung Health Unit, Melbourne School of Population and Global Health, The University of Melbourne, Victoria, Australia; 8 Centre for Neuropsychiatric Schizophrenia Research (CNSR) & Centre for Clinical Intervention and Neuropsychiatric Schizophrenia Research (CINS), Mental Health Centre Glostrup, University of Copenhagen, Glostrup, Denmark; 9 Section of Food, Microbiology and Fermentation, Department of Food Science, University of Copenhagen, Copenhagen, Denmark; 10 Child and Adolescent Mental Health Centre, Copenhagen University Hospital - Mental Health Services CPH, Copenhagen, Denmark; 11 PsychGen Centre for Genetic Epidemiology and Mental Health, Norwegian Institute of Public Health, Oslo, Norway; 12 Psychiatric Genetic Epidemiology Group, Research Department, Lovisenberg Diaconal Hospital, Oslo, Norway; 13 Population Health Sciences, Bristol Medical School, University of Bristol, Bristol, UK

**Keywords:** ADHD, Genetics

## Abstract

In observational studies, an unhealthy dietary pattern during pregnancy is associated with an increased likelihood of offspring ADHD. We investigated whether such associations may be partly attributable to genetic confounding. Polygenic scores (PGSs) for a healthy dietary pattern were calculated for mother, father, and child trios in the COPSAC_2010_ cohort. Diagnoses and trait scores of ADHD were assessed in the children at age 10. Using trio models, we tested whether the association between maternal genetic liability to a healthy dietary pattern and offspring ADHD could be accounted for by genetic confounding. In COPSAC_2010_ trio models (N-trio=437), a maternal healthy dietary pattern PGS was associated with reduced ADHD trait score after adjustment for child and paternal dietary pattern PGS, suggesting indirect genetic effects consistent with causal effects from maternal diet. However, this was not replicated in MoBa (N-trio=41 580) or ALSPAC (N-trio=1 211), where direct genetic effect estimates implied an important role for genetic confounding. Collectively, these genetic results indicate a potential pathway by which genetic confounding can inflate observed associations between maternal dietary pattern in pregnancy and offspring ADHD, and do not provide any robust evidence consistent with a causal pathway between the two. These findings should be interpreted in the light of both the limited predictive power of the dietary pattern PGS, which accounted for only a small proportion of pregnancy diet variance, and the multi-faceted nature of diet as an exposure.

## Introduction

Attention deficit hyperactivity disorder (ADHD) is a common neurodevelopmental disorder reported to affect 5.9-7.1% of children and adolescents [1]. It is well established that most of the variability in liability to ADHD is driven by a combination of genetic predisposition and early environmental exposures, where especially the intrauterine environment is believed to play a pivotal role [2]. Questions concerning the causality of these pregnancy-related environmental associations with ADHD have recently emerged, as their links with ADHD may be confounded by shared familial factors such as maternal genetic liability [3–5]. For example, smoking in pregnancy has been associated with a twofold incidence of ADHD in large population-based studies [2, 6, 7]. However, using genetically informed study designs including sibling designs, which can account for both shared environment and genetic predisposition, this association has been consistently shown to be driven by familial confounding. This means that smoking in pregnancy is not likely a causal predisposing factor for offspring ADHD, but rather that a genetic predisposition to ADHD increases the likelihood of being exposed to smoking in utero [2, 3, 8, 9]. This example shows the importance of accounting for genetic predisposition which as a classical confounder may induce non-causal associations.

Fetal brain development is a rapid and complex process with a high vulnerability to insufficient nutrient supply, consequences of which may include increased susceptibility to neurological disorders [10]. There is emerging evidence from observational studies [11–13] and pre-clinical animal models [11, 14] that an unhealthy dietary pattern during pregnancy may increase the likelihood of child neurodevelopmental conditions, including ADHD and higher load of ADHD traits. Potential mechanisms include the pro-inflammatory properties of a western diet and disrupted lipid metabolism [11, 14, 15]. Inferring causality from observational associations between maternal diet and offspring ADHD is challenging due to the strong genetic component of ADHD, with heritability estimates as high as 80% [3, 16]. Maternal genetic predisposition to ADHD likely confounds these associations by influencing both dietary intake in pregnancy and an increased likelihood of ADHD in the child. In the Copenhagen Prospective Studies on Asthma in Childhood (COPSAC) mother-child cohorts, we previously identified an association between a Western dietary pattern (high intake of animal fats, refined grains, high fat dairy, red meat, processed meat) during pregnancy and an increased risk of offspring ADHD. This finding was replicated across three independent mother-child cohorts, strengthening the validity of the association. Importantly, the relationship persisted after rigorous confounder adjustment, including maternal polygenic scores (PGSs) for ADHD to partially account for genetic predisposition [15]. Family-based study designs as the genetic trio model offer an alternative and more robust methodological approach to disentangle genetic and environmental effects, which may be a step towards identifying true causal relationships and inform the design of randomized controlled trials (RCTs) and potential intervention strategies [17]. With growing knowledge about the genetic underpinnings of dietary patterns [18], it is now possible to calculate PGSs that reflect individual predisposition to specific dietary behaviours. A large-scale genome-wide association study (GWAS) based on Food Frequency Questionnaires (FFQs) found a considerable heritable component to dietary intakes, where common genetic variants explained 13.6% of the variance for a FFQ-derived dietary pattern [18]. This suggests that the observed association between maternal diet and offspring ADHD may be partially due to underlying genetics conferring a tendency to have an unhealthy diet and also increased likelihood of ADHD, which is then passed on to the child, so-called “genetic confounding”.

**Fig. 1:**
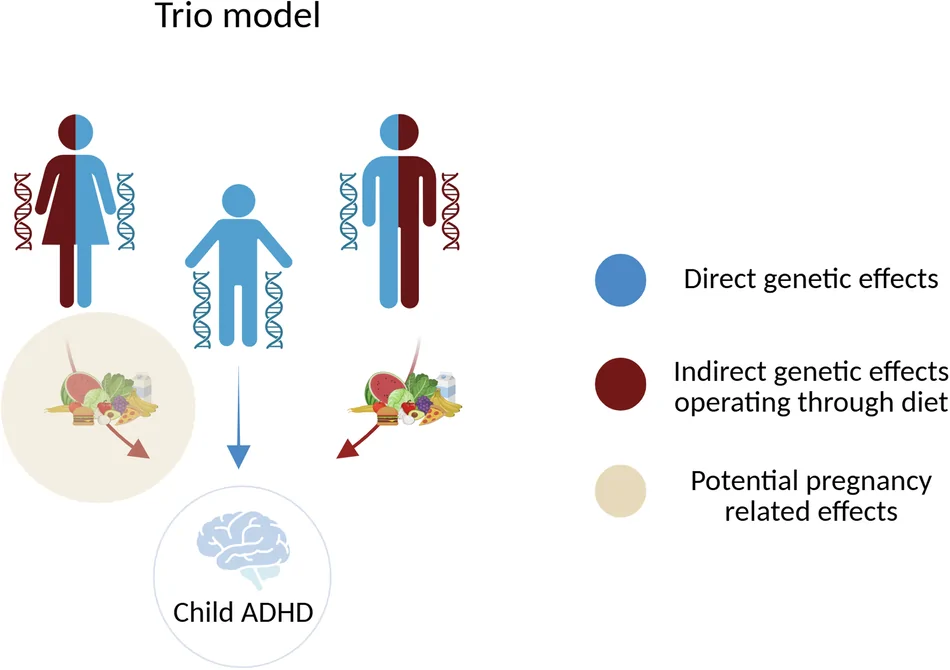
Graphical presentation of the trio polygenic score model to estimate direct and indirect genetic effects of diet in pregnancy on offspring likelihood of attention deficit hyperactivity disorder. The figure was drawn based on a directed acyclic graph of the trio model by Pingault et al. [19]. Created in BioRender. Bønnelykke, K. (2026) https://BioRender.com/ghuyslo.

Genetic trio models, incorporating both parents’ and child’s genetic liability to a given phenotype that corresponds to the environmental exposure of interest, can be used to decompose associations between parental exposures and child outcomes, and overcome some limitations of studies using sibling-pairs or adopted children [19]. Trio models leverage the fact that parents can transmit genetic disposition for a disorder to the child, both directly through inherited alleles and indirectly through non-transmitted alleles that influence the environment surrounding the child (Fig. 1) [20, 21]. Evidence of direct genetic effects implies that an observational association between the same traits is subject to “genetic confounding”, whereas indirect genetic effects including mechanisms operating through environmental pathways, as such, are consistent with causality underpinning observed associations. Trio models have previously been used to examine suggested predisposing factors for ADHD, including psychopathological conditions, smoking, alcohol use, and educational attainment. Using genetic trio models for these factors, direct genetic effects were predominant, and indirect genetic effects were demonstrated only for maternal neuroticism [19].

Here, we use genetic trio models to investigate dietary patterns in pregnancy as a predisposing factor for offspring ADHD. The goal of the study is to estimate the likely role of genetic confounding in explaining observed links between maternal diet in pregnancy and offspring ADHD. To the extent that trio models reveal direct effects of children’s genetic liability to particular dietary patterns on their ADHD symptoms and likelihood of receiving ADHD diagnoses, this will indicate a genetic confounding pathway in observational associations. In contrast, maternal indirect genetic effect estimates from the trio models - where mothers’ genetic liability to specific dietary patterns independently predicts offspring ADHD outcomes - are consistent with the possibility of a causal mechanism underpinning the observed links between the two. To strengthen the validity and generalizability of our findings, we sought external replication in two large independent family cohorts; the Norwegian, Mother, Father and Child Cohort Study (MoBa) and the Avon Longitudinal Study of Parents and Children (ALSPAC).

## Methods

### Study population

This project was set in the COPSAC_2010_ cohort, which includes a population-based sample of 700 mother-child pairs. Pregnant women were recruited to the cohort between November 2008 and November 2010. Mothers were followed at the COPSAC research unit from week 24 of pregnancy, and the offspring with 14 clinical visits from birth until the age of 10, where the COpenhagen Prospective Study on Neuro-PSYCHiatric Development (COPSYCH) neurodevelopmental evaluation was conducted [22, 23]. In the current study, we excluded one individual from each of the 10 total set of twins in the COPSAC_2010_ cohort and removed genetically determined non-biological parents. The study was conducted in accordance with the guiding principles of the Declaration of Helsinki and was approved by the Local Ethics Committee (H-B-2008-093, and the Danish Data Protection Agency (2015-41-3696). Both parents gave written informed consent before enrolment.

We sought replication of findings from the COPSAC_2010_ cohort using data from the MoBa and ALSPAC cohorts.

The Norwegian MoBa study is a population-based pregnancy cohort study conducted by the Norwegian Institute of Public Health. Participants were recruited from all over Norway from 1999-2008. The women consented to participation in 41% of the pregnancies (Informed Consent) [24]. The cohort includes approximately 114,500 children, 95,200 mothers, and 75,200 fathers. MoBa is regulated by the Norwegian Health Registry Act. Blood samples were obtained from both parents during pregnancy and from mothers and children (umbilical cord) at birth [25]. The current project uses information on child sex and birth year from the Medical Birth Registry (MBRN) which is a national health registry containing information about all births in Norway.

ALSPAC recruited pregnant women from the Southwest of England, Avon, with an expected delivery between April 1991 and December 1992. Of all eligible pregnancies, 14,541 pregnant women, corresponding to 72%, consented to participate (13,988 alive at age one) (Informed Consent). An additional enrollment of pregnancies resulted in a sample size of 15,447 pregnancies for data collected after the age of seven. Of these, 14,901 children were alive at 1 year of age [26, 27]. G0 partners (partners of the original cohort) were invited to complete questionnaires by the mothers at the start of the study and they were not formally enrolled at that time. 12,113 G0 partners have been in contact with the study by providing data and/or formally enrolling when this started in 2010. 3,807 G0 partners are currently enrolled [28]. Please note that the study website contains details of all the data that is available through a fully searchable data dictionary and variable search tool [29]. Ethical approval for the study was obtained from the ALSPAC Ethics and Law Committee and the Local Research Ethics Committees.

In both MoBa and ASLPAC one twin from each set was kept. (Supplementary Methods)

### Polygenic scores for dietary pattern

The PGS for dietary pattern was based on a GWAS by Cole et al. on 449,210 individuals of European ancestry with dietary information from FFQs [18]. The GWAS analyzed principal component analysis (PCA) derived dietary patterns, where the dietary pattern PGS in the present study was calculated according to the first principal component (PC1) which was the highest heritable dietary pattern (heritability estimate = 0.136) and had the most genome-wide significant loci (significant GWAS loci = 140). PC1 explained 8.63% of the phenotypic variance in curated measures of single food intake and was defined by foods that have been described in the literature as Western and prudent dietary factors [18]. Significant positive loadings for PC1 included increased intake of wholegrain bread, fruits and vegetables, oily fish, and water (a “healthy” dietary pattern). Significant negative loadings included intake of white bread, butter and oil spread, processed meat, and high-fat milk (an “unhealthy” dietary pattern) [18, 30].

PGSs were constructed using PRS-CS for the COPSAC_2010_ and ALSPAC cohorts (Supplementary Methods). For MoBa, the PGS was constructed using the comparable LDpred2 method and was adjusted for genotyping and imputation batch and population structure (first 20 PCs) [31, 32]. Details on the genotyping of MoBa and ALSPAC trios are available elsewhere [33, 34]. Reported associations are for 1 SD increase in the PGS corresponding to genetic predisposition to eating a healthier dietary pattern.

### Validation of dietary pattern polygenic scores

We tested whether the dietary pattern PGS predicted reported consumption of similar food items in pregnant women from the COPSAC_2010_ cohort as the PC1 described by Cole et al. [18]. This was done by plotting the maternal PGS against the intake of 43 food items reported in a validated semi-quantitative FFQ by mothers in week 24 of pregnancy [35]. The predictive value of the dietary pattern PGS was further tested by its correlation to a maternal pregnancy Western dietary pattern derived from a PCA based on energy, macronutrient, and micronutrient intake, also calculated from FFQs, which was previously associated with offspring neurodevelopmental disorders in COPSAC_2010_ [15]. We also examined the effect of the dietary pattern PGS on the 20 food groups most strongly associated with the Western dietary pattern, and conversely, the effect of the Western pattern on those most strongly linked to the PGS. Lastly, we calculated mean Western dietary pattern PC for 2 SD intervals of the maternal dietary pattern PGS.

Dietary pattern PGSs were likewise validated in the replication cohorts using FFQ data. In MoBa, a 255-item validated FFQ was administered at gestational week 22, covering maternal dietary habits since she became pregnant [36–39]. FFQ items were sorted into intake of 98 food groups measured by grams per day [40]. In ALSPAC, the FFQ was administered at week 32 of pregnancy and covered the current intake of the 43 most frequently consumed foods in the UK. More detailed questions were asked for an additional 8 foods usually consumed daily, which enabled us to include the estimated weekly intake of wholemeal and white bread. A healthy pregnancy dietary pattern PC based on the FFQ data (explaining 10.6% of variance) with high loadings of, among others, salad, fruit, oat and bran-based cereals, fish, and non-white bread, was likewise used for validation of the dietary pattern PGS in the ALSPAC cohort [41].

Finally, to estimate the explained variation in pregnancy dietary intake in alle three cohorts by maternal dietary pattern PGS, we used FFQ data to derive dietary pattern PC’s. (Supplementary Methods)

### Neurodevelopmental outcomes

At the age of 10 years the COPSAC_2010_ cohort underwent an in-depth clinical psychopathological evaluation where the study’s main outcomes, diagnoses of ADHD and ADHD trait scores, were assessed.

Child and parent(s) were interviewed separately with the semi-structured clinical diagnostic interview Kiddie Schedule for Affective Disorders and Schizophrenia Present and Lifetime Version (K-SADS-PL) by trained interviewers, including medical doctors, nurses, and psychologists [42]. Research diagnoses were based on a collective evaluation of the K-SADS-PL and all other available clinical information and assigned according to the International Classification of Disorders 10^th^ revision (ICD-10) [43]. ADHD was defined by the diagnostic codes DF90.0, DF90.8, and DF98.8 C. We grouped diagnoses according to DSM-5 terminology with ADHD Combined Presentation including DF90.0 and DF90.8, and ADHD Inattentive Presentation including DF98.8 C. (Supplementary Methods)

ADHD trait scores from all included cohorts referred to the 18 DSM-IV items for ADHD, with 9 items for inattentive and 9 items for hyperactivity/impulsivity symptoms. For all scales, responses were made on a 4-point Likert scale. Scores were not transformed in analyses.

In COPSAC_2010_, ADHD trait score was assessed by the ADHD Rating Scale (ADHD-RS) filled out by parents [44].

Neurodevelopmental outcomes in MoBa included child ADHD diagnoses obtained from the Norwegian Patient Registry (NPR). ADHD was defined by at least one registered ICD-10 diagnostic code of hyperkinetic disorder including DF90.0, DF90.1, DF90.8, DF90.9, and DF98.8 regardless of age at registration. Combined Presentation included DF90.0, DF90.1, DF90.8, DF90.9 and Inattentive Presentation included DF98.8. The prevalence of ADHD diagnoses from the NPR after the exclusion of one twin from each pair was 7,5% (N ADHD = 8,305). Mother reported traits of ADHD were assessed at the age of 8 by the Parent Rating Scale for Disruptive Behaviour Disorders (RS-DBD) [45].

In ALSPAC, mother-reported ADHD traits were evaluated using the structured diagnostic interview Development and Wellbeing Assessment (DAWBA) at the age of 7 [46, 47]. (Supplementary Methods)

### Statistical analysis

We used linear regression and quasi-Poisson models to test the ability of the dietary pattern PGS to predict food intake measured by FFQ. The Pearson correlation was tested between dietary pattern PGS and PC-derived pregnancy Western dietary pattern and maternal ADHD PGS [48]. Additionally, we tested the genetic correlation using linkage disequilibrium score regression (LDSC) between maternal dietary pattern genetics and ADHD genetics [49].

In the main analyses, the associations between maternal, child, and paternal dietary pattern PGS and ADHD outcomes were tested using logistic regression for ADHD diagnosis and linear regression for ADHD total trait scores. Sensitivity analyses were performed with ADHD presentations and ADHD trait subscales as outcomes. All trio models were based on a single regression model including maternal, paternal, and child dietary pattern PGSs as predictors of offspring ADHD, with each PGS mutually adjusted for the others [19]. In follow-up analyses, we evaluated the effect of the dietary pattern PGS in the general population by performing trio models on ADHD trait scores within individuals without an ADHD diagnosis. Further, we performed trio models on the dietary pattern PGS adjusted for the matching ADHD-PGSs to separate the effects of dietary genetics from ADHD genetics [48]. All analyses were adjusted for the sex of the child. Trio models indicating indirect genetic effects were rerun with adjustment for social circumstances variables and putative prenatal predisposing factors for ADHD to adjust for measured confounding through traits related to dietary pattern genetics. All potential confounders were included in this model regardless of the association to maternal dietary pattern PGS to minimize residual confounding. We sought replication using a similar statistical approach in the MoBa and ALSPAC cohorts. Trio models on MoBa data were additionally adjusted for birth year to account for the longer inclusion time. Additional sensitivity analyses were performed for MoBa; trio models on ADHD diagnosis were performed, requiring a minimum of two registered ADHD diagnoses in NPR, and further trio models were performed using clustered robust standard errors based on maternal ID to account for the inclusion of siblings.

All statistical tests were performed with an alpha of 5%. Data was analyzed using the statistical software R, version 4.4.1. Analytic code for the project is available at https://github.com/psychgen/preg-diet-neurodev.

## Results

### Baseline characteristics

In COPSAC_2010_, a total of 593 individuals were clinically evaluated for neurodevelopmental and other psychiatric disorders at age 10 years, and an additional 11 individuals had questionnaire-based information on symptom loads. 530 had data on diet PGS for at least one family member and 437 had complete trio information (Supplementary Figure 1). Cohort demographics are described according to diagnosis of ADHD in Table 1. Among complete trios 12.1% were classified with ADHD as compared to 11.0% among the total 593 evaluated children. A drop-out analysis comparing complete trios to the remaining cohort showed a higher degree of participation in the fish oil intervention and a marginally higher mean Western dietary pattern PC among complete trios (Supplementary Table 1).

**Table 1:** Baseline characteristics of COPSAC_2010_ families with complete trio information, stratified according to diagnosis of ADHD.

	No ADHD	ADHD	P value
	n = 384	n = 53	
Child dietary pattern PGS, mean (SD)	0.12 (1.00)	-0.43 (1.11)	<0.001
Maternal dietary pattern PGS, mean (SD)	-0.04 (1.05)	-0.36 (0.91)	0.037
Paternal dietary pattern PGS, mean (SD)	0.06 (0.99)	-0.18 (0.92)	0.090
Sex, male, n (%)	180 (46.9)	41 (77.4)	<0.001
Gestational age, d, mean (SD)	279.17 (11.16)	280.49 (8.66)	0.285
Birthweight, kg, mean (SD)	3.54 (0.53)	3.64 (0.48)	0.122
Maternal educational level, n (%)			0.005
Elementary school	10 (2.6)	4 (7.5)	
College graduate	20 (5.2)	4 (7.5)	
Tradesman certification	67 (17.4)	18 (34.0)	
Bachelors’ degree	173 (45.1)	19 (35.8)	
Masters’ degree or higher	114 (29.7)	8 (15.1)	
Paternal educational level, n (%)			0.093
Elementary school	14 (3.7)	3 (5.7)	
College graduate	26 (6.8)	0 (0.0)	
Tradesman certification	124 (32.4)	25 (47.2)	
Bachelors’ degree	111 (29.0)	12 (22.6)	
Masters’ degree or higher	108 (28.2)	13 (24.5)	
Household income, n (%)			0.286
<100.000 DKK	30 (7.8)	3 (5.7)	
100.000-150.000 DKK	90 (23.4)	17 (32.1)	
150.000-200.000 DKK	116 (30.2)	16 (30.2)	
200.000-250.000 DKK	87 (22.7)	13 (24.5)	
>250.000 DKK	61 (15.9)	4 (7.5)	
Alcohol intake in pregnancy, yes, n (%)	60 (15.6)	6 (11.5)	0.397
Smoking in pregnancy, yes, n (%)	24 (6.2)	6 (11.3)	0.195
Parity, n (%)			0.269
1	171 (44.5)	20 (37.7)	
2	159 (41.4)	22 (41.5)	
≥3	54 (14.1)	11 (20.8)	
Maternal pre-pregnancy BMI, mean (SD)	24.53 (4.28)	26.43 (5.66)	0.004
Maternal Western dietary pattern PC, mean (SD)	-0.02 (0.93)	0.51 (1.07)	0.001
Maternal age, y, mean (SD)	32.05 (4.13)	32.03 (4.61)	0.671
Paternal age, y, mean (SD)	34.52 (5.14)	34.54 (5.10)	0.681
Vitamin D intervention, yes, n (%)	152 (47.8)	23 (50.0)	0.627
Fish oil intervention, yes, n (%)	209 (54.6)	24 (45.3)	0.181

*ADHD* attention deficit hyperactivity disorder, *PGS* polygenic score, *BMI* body mass index.

### Validation of the dietary pattern polygenic score

**Fig. 2:**
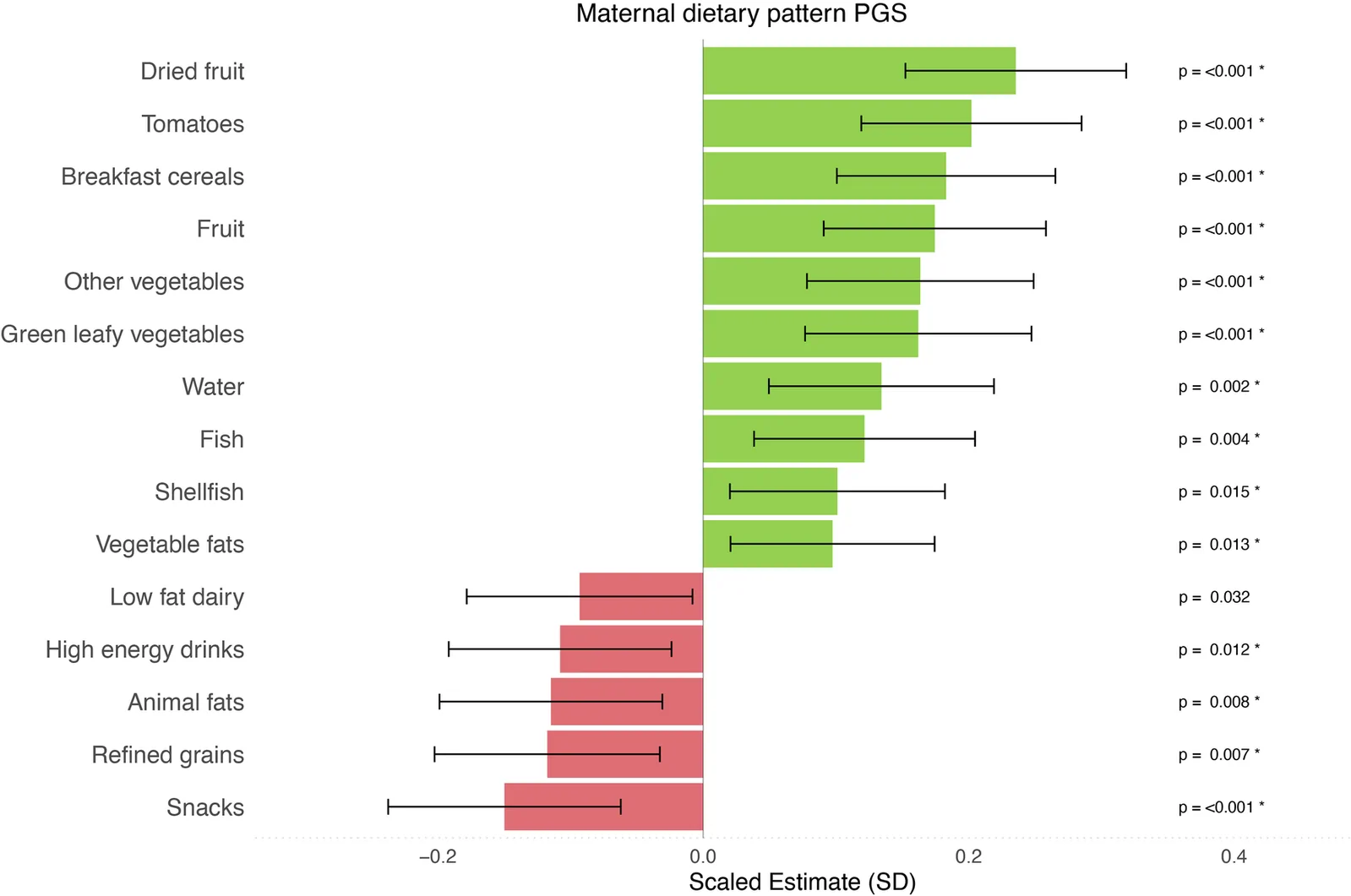
Maternal dietary pattern polygenic score plotted against maternal diet as reported by food frequency questionnaires in week 24 of pregnancy. Only nominal significant associations are shown. FDR significant p-values are marked with an asterisk (*).

The maternal dietary pattern PGS showed an association to maternal diet in pregnancy in COPSAC2010 comparable to findings of Cole et al. [18]. where the diet PGS was generated (Fig. 2) and showed a significant correlation with the PC derived Western dietary pattern in pregnancy previously associated with neurodevelopmental disorders in COPSAC_2010_ (Pearson’s r = -0.28; 95%CI = -0.36 – -0.20, *p* < 0.001) [15]. Top food groups associated with the maternal dietary PGS were associated in a similar pattern to the Western dietary pattern PC. When depicting top food groups for the Western dietary pattern PC, the maternal dietary pattern PGS also showed similar directionality of associations (Supplementary Figures 2-3). Mean maternal dietary pattern PC decreased with higher values of the maternal dietary pattern PGS (Supplementary Figure 4). The maternal dietary PGS was associated with maternal smoking in pregnancy, maternal and paternal education, but not with other strong ADHD correlates such as maternal pregnancy C-reactive protein (CRP) [50] and maternal pre-pregnancy body mass index (BMI) (Supplementary Table 2) [51]. Maternal and paternal dietary pattern PGSs were not correlated (r = 0.04; 95%CI = -0.05–0.12, *p* = 0.406). For validation cohorts, the validity of the maternal dietary pattern PGS was tested on pregnancy FFQ data. In MoBa, 83 of the included 98 food groups were significantly associated with the maternal dietary pattern PGS after FDR correction. Consistent with the pattern described by Cole et al., a higher PGS positively associated with intake of olive oil, vegetables, water, wholegrain cereals, and brown bread, and negatively associated with intake of sugar sweetened soft drinks, processed meat, white bread, chips, and animal fats (Supplementary Figure 5). In ALSPAC, maternal PGS was positively associated with the intake of pudding, wholegrain cereals, whole meal bread, juice, and negatively associated with the intake of white bread and roast potatoes (Supplementary Figure 6), and further positively correlated with maternal healthy diet PC (Pearson’s r = 0.04 p-value 0.001).

Using FFQ data from all three cohorts to derive pregnancy dietary pattern PCs, only results from COPSAC showed similar degrees of explained variance as Cole et al. (Supplementary Table 3 and Supplementary Figures 7-9).

### Dietary and ADHD genetic overlap

Maternal dietary pattern PGS showed a weak correlation to maternal ADHD PGS (Pearson’s r = -0.10; 95%CI = -0.18– -0.02, *p* = 0.015) in the COPSAC_2010_ cohort, and there was a modest genetic correlation between dietary pattern genetics and ADHD genetics based on summary statistics from published GWAS (r = -0.24; SE 0.04, *p* < 0.001).

### Dietary pattern PGS and ADHD outcomes in the COPSAC_2010_ cohort

Without adjustment for any other family members dietary pattern PGSs, a one standard deviation increase in maternal dietary pattern PGS, corresponding to a higher genetic predisposition to a healthy diet, was associated with a 29% lower likelihood of ADHD diagnosis (OR = 0.71; 95%CI = 0.53–0.93, *p* = 0.015). Paternal dietary pattern PGS showed a similar effect size (OR = 0.77; 95%CI = 0.57–1.03, *p* = 0.085). Child dietary pattern PGS had the strongest association with ADHD diagnosis (OR = 0.55; 95%CI = 0.41–0.73), *p* < 0.001). Higher maternal and child dietary pattern PGS were also associated with lower offspring ADHD-RS total score (mother beta = -1.23; 95%CI = -1.93–-0.53, *p* < 0.001; child beta = -1.10; 95%CI = -1.82–-0.38, *p* = 0.003). No association was observed between paternal dietary pattern PGS and ADHD-RS total score (beta = -0.50; 95%CI = -1.30-0.29, *p* = 0.212). (Supplementary Table 4)

### Trio analyses in the COPSAC_2010_ cohort

The association between maternal and paternal dietary pattern PGS and offspring ADHD diagnoses attenuated after adjustment for direct genetic transmission in the trio models (mother OR = 0.98; 95%CI = 0.69–1.38, *p* = 0.893; father OR = 1.05; 95%CI = 0.73–1.53, *p* = 0.780), while the child dietary pattern PGS association remained consistent (child OR = 0.58; 95%CI = 0.38–0.88, *p* = 0.011). This indicates that direct genetic effects may contribute to observational associations between diet in pregnancy and ADHD diagnosis.

**Fig. 3:**
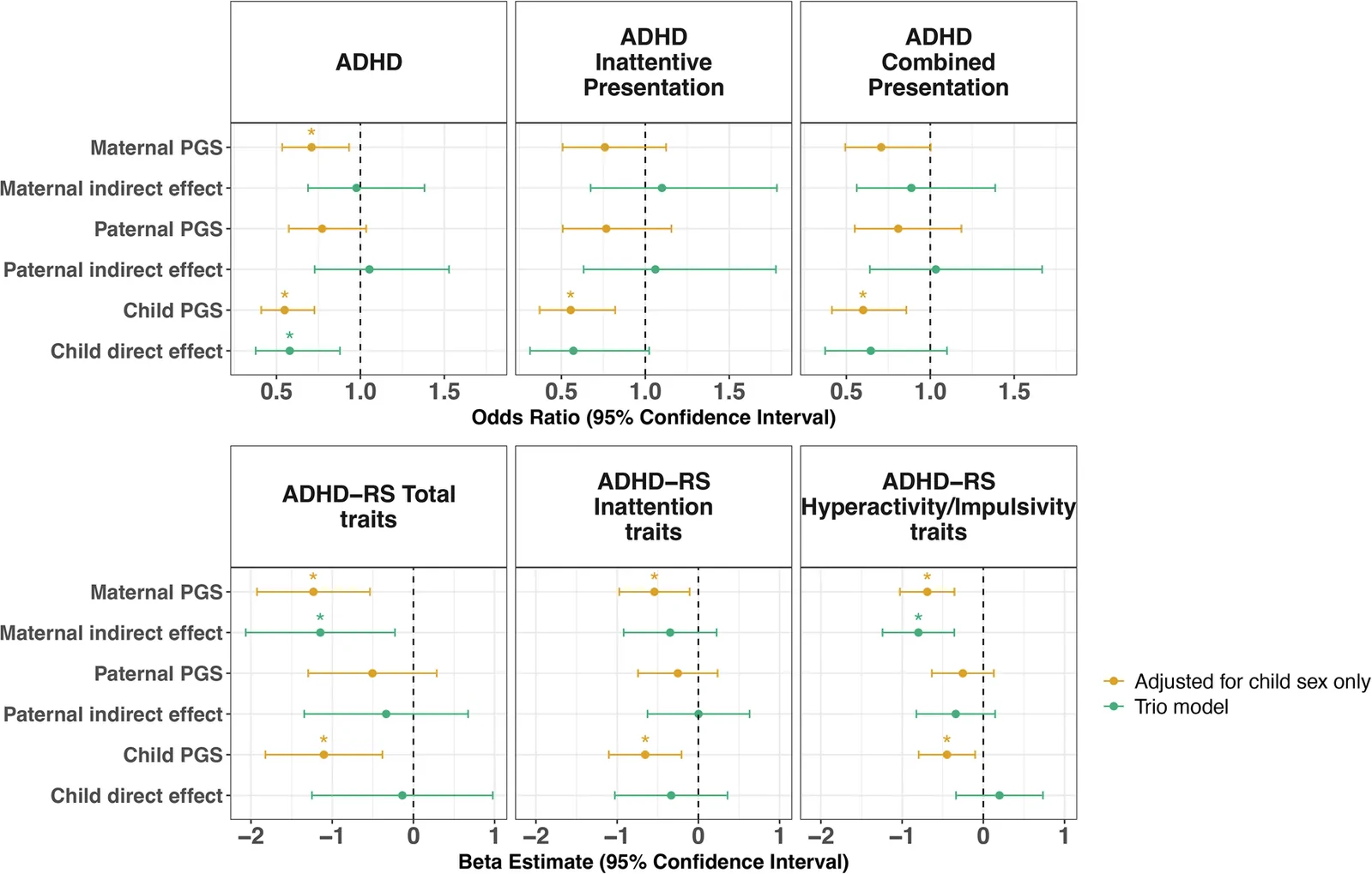
Unadjusted and trio models of the association between dietary pattern PGS and ADHD K-SADS-PL diagnosis as well as ADHD-RS trait score in the COPSAC_2010_ cohort. Unadjusted analyses were performed without adjustment for any other family members dietary pattern PGSs. Trio models were based on a single regression model including maternal, paternal, and child dietary pattern PGSs as predictors of offspring ADHD, with each PGS mutually adjusted for the others. All analyses were adjusted for sex of the child.

In contrast, there was evidence of maternal indirect genetic effects on ADHD trait score (mother beta = -1.15; 95%CI = -2.06 – -0.23, *p* = 0.015) and no evidence of direct genetic effects, with the child dietary pattern PGS estimate attenuating in trio models for this outcome (child beta = -0.14; 95%CI = -1.25-0.98, *p* = 0.810). No paternal indirect genetic effects on ADHD trait scores were observed, suggesting that the observed effects are exclusively maternal (father beta = -0.34; 95%CI = -1.35–0.68, *p* = 0.514) [52]. In analyses on ADHD-RS subscales, there was evidence of indirect genetic effects also for Impulsivity and Hyperactivity traits, but not for Inattentive traits. (Fig. 3 & Supplementary Table 5)

Exclusion of ADHD cases and adjustment for ADHD PGS in trio models did not alter the results (Supplementary Figure 10 & 11). The maternal indirect genetic effect on ADHD trait score was attenuated after adjustment for social circumstances variables and putative predisposing factors for ADHD including maternal pregnancy week 24 CRP, maternal pre-pregnancy BMI, maternal age, paternal age, household income, maternal and paternal educational level, sex, birth weight, gestational age, and fish oil supplementation in third trimester: adjusted beta = -0.86; 95%CI = -1.76–0.05, *p* = 0.064. (Supplementary Results)

### Replication in the MoBa and ALSPAC cohorts

**Fig. 4:**
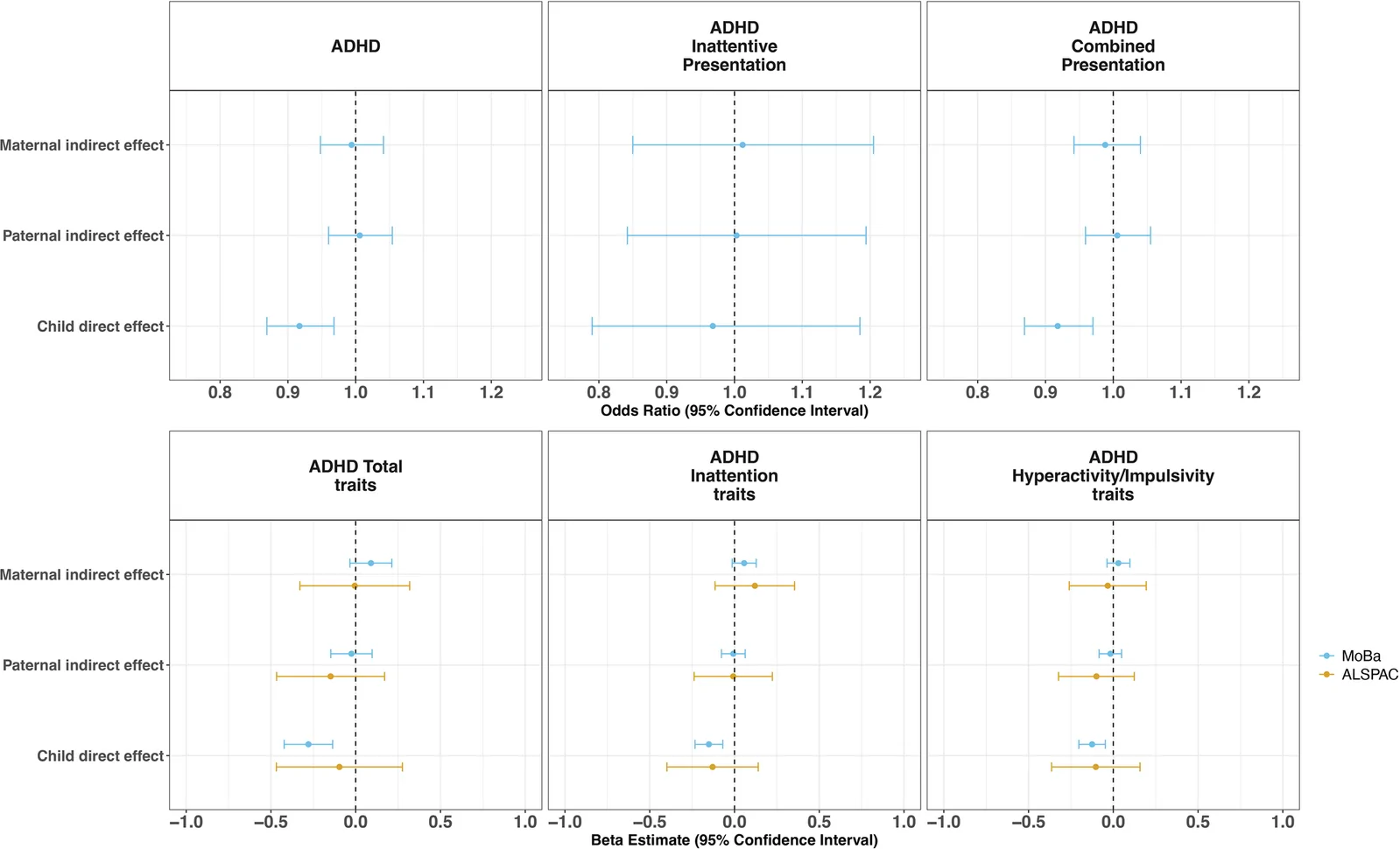
Trio models of the association between dietary pattern PGS and ADHD diagnosis as well as ADHD trait score for MoBa and ALSPAC. ALSPAC analyses were adjusted for sex of the child and MoBa analyses included additional adjustment for birth year due to the longer period of inclusion.

Analyses using data from the MoBa cohort included 41,580 complete trios in analyses on ADHD diagnoses (ADHD prevalence 7,0%) and 18,629 complete trios for ADHD symptoms (median RS-DBD total score 7 (IQR 4-11). Child, maternal, and paternal dietary pattern PGSs for ADHD were all associated with ADHD diagnosis and ADHD total trait score in unadjusted models. In trio analyses, there was no evidence of indirect genetic effects (maternal OR for ADHD = 0.99; 95%CI = 0.95–1.04, *p* = 0.784, maternal beta for ADHD traits = 0.09; 95%CI = -0.03–0.21, *p* = 0.156), and only child dietary pattern PGS remained associated with ADHD outcomes indicating direct genetic transmission of risk only (child OR for ADHD = 0.92; 95%CI = 0.87–0.96, *p* = 0.002, child beta for ADHD traits =-0.28; 95%CI = -0.42– -0.14, *p* < 0.001). Results from 1211 complete trios (median total DAWBA score 2 (IQR 0-7)) from the ALSPAC cohort were consistent in direction and magnitude, though less precisely estimated, meaning that no effects were different from zero (Fig. 4, Supplementary Results, Supplementary Figures 12-13, and Supplementary Tables 6-9).

## Discussion

We used genetic trio models to investigate potential mechanisms underpinning observed associations between maternal dietary pattern in pregnancy and ADHD in the offspring. Overall, trio models applied across three cohorts indicated a likely role for genetic confounding in observational associations between maternal dietary pattern and offspring ADHD diagnosis, with consistent evidence for direct effects from transmitted genetic liabilities to specific dietary patterns on the likelihood of receiving the diagnosis among offspring. Our results overall did not provide support for a causal link between maternal dietary pattern and offspring ADHD diagnoses, with a lack of evidence of effects of maternal dietary pattern PGS after control for genetic liability directly transmitted to offspring. Trio models in the COPSAC_2010_ cohort showed evidence for indirect genetic effects that would be consistent with a causal relationship between maternal diet and offspring ADHD traits. However, no similar effects were observed in the larger external replication cohorts MoBa and ALSPAC.

To our knowledge, this is the first study to explore genetic confounding in links between dietary patterns and offspring ADHD using a genetic trio model approach [12]. By using a healthy dietary pattern PGS, we showed evidence of direct genetic transmission, suggesting that previously reported observational findings may be impacted by genetic confounding. Genetic confounding describes a scenario where an observational association is wholly or partly attributable to the fact that the same genetic factors influence both the exposure and outcome. Existing studies have to some extent accounted for this. In the COPSAC_2010_ cohort, the substantial increase in likelihood of ADHD following a Western prenatal dietary pattern was robust to adjustment for maternal ADHD PGS and was replicated across several mother-child cohorts [15]. The study further found that ADHD genetics moderated the effect of the Western dietary pattern supporting the role of genetics for such associations [15]. An observational study from the MoBa cohort reported small, though robust, associations between quality of pregnancy diet and offspring likelihood of ADHD diagnoses and ADHD traits across strata of registered maternal ADHD traits in an attempt to account for maternal ADHD genetic disposition [13]. Using the largest available number of genotyped trios, we did not find evidence in our genetic-based models that would be consistent with effects of diet in pregnancy, as defined by a healthy dietary pattern PGS, on either ADHD diagnosis nor number of ADHD traits, including individuals not reaching the diagnostic threshold for ADHD. Despite the inclusion of a high number of complete trios, the study may have been underpowered to detect indirect effects. The heritability of the Cole et a. dietary pattern PC1 was 13.6%. Using birth cohort data to generate matching PCs, the variance explained was 10% in COPSAC but smaller for MoBa (3%) and ALSPAC (0%) which may have substantially limited our ability to detect evidence consistent with causal effects of diet. However, indirect effects of maternal neuroticism on offspring ADHD have been shown using a PGS explaining 4% of variance [19], and direct effects of maternal smoking on offspring ADHD and conduct problems using a PGS explaining <1% [19, 53–55].

We found some evidence consistent with a causal effect of diet on ADHD trait score in the COPSAC_2010_ cohort, which was not replicated in MoBa or ALSPAC despite larger study numbers. This ability to detect indirect effects in COPSAC could be explained by a more precise assessment of ADHD traits as compared to MoBa and ALSPAC. The UK Biobank GWAS from which the dietary pattern PGS was derived reported a significant genetic correlation with factors not related to diet, including physical activity, educational attainment, socioeconomic status, and smoking status. For BMI, the study reported that there may be a shared genetic etiology but that the underlying genetic architectures are predominantly distinct [18]. The indirect genetic effect in COPSAC_2010_ trio analyses may be a product of confounding from such lifestyle factors related to both ADHD and dietary pattern genetics, and the attenuation of the effect after adjustment for lifestyle factors indicated that this could be the case. Further, the maternal and paternal dietary pattern PGS predicted ADHD similarly across cohorts, whereas a true causal effect of maternal dietary pattern would be expected to result in relatively stronger prediction from maternal PGS. Finally, we consider residual confounding to be an unlikely explanation for the lack of indirect estimates in MoBa and ALSPAC as lifestyle factors would be expected only to inflate indirect effects by their positive association to both an unhealthier diet and risk of ADHD.

The trio design is a major strength of this study. To investigate the observational associations between pregnancy diet and ADHD further, results should be sought triangulated across different study designs with different sources of bias. For this purpose, genetically informed studies such as ours are an important contribution, due to the design-based handling of confounding which is believed to be a strong complementary approach to traditional statistical adjustment [17]. The reliability of our findings is significantly substantiated by the ability to validate the dietary pattern PGS derived from summary statistics based on a non-pregnant population in all three included cohorts using FFQ data. Though the COPSAC_2010_ cohort includes the smallest number of trios compared to MoBa and ALSPAC, the major strength of including analyses within this cohort is the high quality of the phenotypic outcomes based on the thorough COPSYCH clinical examinations, thus avoiding misclassification of a clinician evaluated ADHD diagnosis [23].

Assortative mating and population structure may confound associations from a GWAS and consequently the reported effects in our study. Supportive of a potential bias from assortative mating, we found that parental PGSs were weakly correlated in MoBa and ALSPAC. Bias from assortative mating or population stratification would be expected to inflate indirect genetic effect estimates, but leave estimates of direct genetic effects unchanged [19, 56]. Assortative mating is therefore unlikely to explain the conclusions drawn in this paper. Several limitations relate to the quality of the dietary pattern PGS. The PGS correlated with unexpected food groups (pudding, biscuits, cakes/buns) in the ALSPAC cohort, which brings into question the consistency of the signal captured by the PGS across time and different populations. Further, the PGS was generated based on the intake of a specific dietary pattern primarily driven by type of bread consumed and therefore may not capture effects from specific nutrients [18]. However, even though the PGS was mainly associated with type of bread consumed, it reflected clear patterns of a healthy/unhealthy diet as previously published [15]. The Cole et al. GWAS from where the dietary pattern PGS was derived, was based on a 24-h recall FFQ obtained from an adult population. Data from a GWAS on pregnancy diet from a larger time span was not available, however, such data could have increased the predictive value of the PGS. Finally, the study was restricted to individuals of European ancestry, which limits the generalizability of our findings.

## Conclusion

In conclusion, using a genetically informative design applied across multiple independent cohorts, we found consistent evidence that genetic liabilities to specific dietary patterns transmitted from parents to children are associated with their ADHD diagnoses and traits – indicating a potential role for genetic confounding in observational links between maternal dietary pattern and offspring ADHD. We did not find strong evidence, in the form of indirect genetic effects in genetic trio models, that would be consistent with a causal mechanism underpinning these links. Important for the overall interpretation, the maternal dietary pattern PGS only explained a limited proportion of the variation in pregnancy dietary intake which despite large study numbers may substantially limit our ability to detect causal indirect effects.

## Supplementary information

Supplementary Information

## Data Availability

Individual-level personally identifiable clinical data from the children participating in the COPSAC cohort cannot be made freely available, to protect the privacy of the participants and their families, in accordance with the Danish Data Protection Act and European Regulation 2016/679 of the European Parliament and of the Council (GDPR) that prohibit distribution even in pseudo-anonymized form. However, research collaborations are welcome, and data can be made available under a joint research collaboration by contacting COPSAC. Requests will be answered within two weeks. Data use is restricted to purposes within childhood health and disease. Information on how to apply for data access for MoBa and ALSPAC data can be found on their respective study websites (URL MoBa: https://www.fhi.no/en/ch/studies/moba/for-forskere-artikler/research-and-data-access/, URL ALSPAC: https://www.bristol.ac.uk/alspac/researchers/access/.

## Governance

We are aware of and comply with recognized codes of good research practice, including the Danish Code of Conduct for Research Integrity. We comply with national and international rules on the safety and rights of patients and healthy subjects, including Good Clinical Practice (GCP) as defined in the EU’s Directive on Good Clinical Practice, the International Conference on Harmonisation’s (ICH) good clinical practice guidelines and the Helsinki Declaration. Privacy is important to us which is why we follow national and international legislation on General Data Protection Regulation (GDPR), the Danish Act on Processing of Personal Data and the practice of the Danish Data Inspectorate.
