# Supplementary material for: Genetic investigation of the association between maternal dietary patterns and offspring ADHD
Source: Mol Psychiatry. 2026 May 25;31(10):5619–28. doi: 10.1038/s41380-026-03645-w (PMC13569445; doi:10.1038/s41380-026-03645-w)
Supplement: Supplementary file 1 [file 41380_2026_3645_MOESM1_ESM.docx]

# Supplementary Information

# **Supplementary methods**

*COPSAC*_2010_ *Study population*

To be included, women were required to speak Danish, consume no more than 600 IU vitamin D during pregnancy; and not suffer from any endocrine, cardiovascular, or nephrological disorders. Two randomized controlled trials were conducted within the cohort, where mothers in their third trimester were randomized to receive supplementation with fish oil and high-dose vitamin D3 in a factorial 2x2 design.

*COPSAC*_2010_ *FFQ data*

The FFQ contained 360 items covering their dietary intake with a one-month recall period. Intake of foods was reported either in natural units or frequency of consumption as appropriate for the given item. Number of grams per day for 43 food groups was calculated based on an assumption of standard portion sizes. Implausible values of energy intake corresponding to below 4,200 kJ/day or above 16,700 kJ/day were excluded.

*COPSAC*_2010_ *neurodevelopmental outcomes*

COPSYCH examinations took place between January 2019 and December 2021. All research diagnoses were assigned in consensus with senior researcher JRJM. An external validation of research diagnoses was performed together with a clinical professor in child and adolescent psychiatry. Video recordings of the clinical examinations allowed for both supervision and testing of interrater reliability which was high. Overall agreement across symptoms currently present and symptoms currently not present was 99.5% CI (99.3-99.7). Agreement of present symptoms was 88.5% CI (82.6-93.0).

*Dietary pattern polygenic scores in COPSAC*_2010_

In the COPSAC_2010_ cohort, high throughput genome-wide single-nucleotide polymorphism (SNP) genotyping of the parents and children was performed using the IlluminaInfinium^TM^ II HumanHap 550K SNP BeadChip technology (Illumina, San Diego) and further by the Illumina Infinium HumanOmniExpressExome BeadChip in the children. We used the PRS-continuous shrinkage (CS) method to construct the score using the 1KG reference panel.^1^ The final score consisted of 1,071,676 SNPs available in both the GWAS summary statistics, reference panel, and the COPSAC cohort. Given the sample size of the GWAS, the phi parameter, controlling the global shrinkage, was estimated based on the data. Each individual SNP effect on diet was updated for each SNP and combined to an aggregated score using the software PLINK(28) and scaled to mean 0 with 1 standard deviation (SD) for each dataset.

*The MoBa study*

The current study is based on Version 12 of the quality-assured data files released for research in January 2019. The establishment of MoBa and initial data collection was based on a license from the Norwegian Data Protection Agency and approval from The Regional Committees for Medical and Health Research Ethics. The MoBa cohort is currently regulated by the Norwegian Health Registry Act.

*MoBa FFQ data*

Implausible values of calorie intake corresponding to an intake of ≥ 4780 (20,000 kJ) or ≤ 1076 kcal (4,502 kJ) were removed. Number of grams per day were calculated for each food group.

*The ALSPAC study*

Consent for biological samples has been collected in accordance with the Human Tissue Act (2004). Informed consent for the use of data collected via questionnaires and clinics was obtained from participants following the recommendations of the ALSPAC Ethics and Law Committee at the time.

*ALSPAC FFQ data*

As previously reported, we converted FFQ data registered with five different frequency options to weekly frequency of intake.^2^ Implausible values of energy intake were excluded based on the MoBa cutoffs.

*ALSPAC neurodevelopmental outcomes*

The ALSPAC DAWBA ADHD trait scores were based on the following variables (See ALSPAC data documentation for further detail): Kr460a indicating total number of inattention/activity symptoms.

Variable 459b and 447b covering prorated scores for attention and activity traits.

*Statistical analysis*

Demographics of the COPSAC_2010_ cohort were reported according to the presence of ADHD, and differences between groups were tested by either t-test or chi-squared test. Associations between maternal dietary pattern PGS and selected social circumstance and prenatal exposure variables were calculated in univariate linear regression models. To test for assortative mating, a potential source of bias in trio models, we calculated the correlation between maternal and paternal dietary pattern PGSs.

We performed principal component analyses (PCA) of the pregnancy FFQ data, to explore the variation in pregnancy FFQ explained by maternal dietary pattern PGS. Prior to conducting the PCA, the intake of each food group was adjusted according to total energy intake. We reported the variation of the FFQ data explained by PC1 and the correlation to maternal dietary pattern PGS.

We did not adjust for multiple testing due to the relatively few main tests performed.

# **Supplementary results**

*Assortative mating in the MoBa and ALSPAC cohorts*

Maternal and paternal dietary pattern PGS were weakly correlated in both the MoBa (Pearson’s r =-0.03, p-value 4.30 × 10^-16^) and ALSPAC cohort (Pearson’s r =-0.05, p-value 0.056).

*MoBa neurodevelopmental outcomes*

After the exclusion of one twin from each set, median number of ADHD RS-DBD traits of ADHD was 7 (IQR 4 -11), and prevalence of ADHD diagnoses from the NPR was 7,5% for all ADHD diagnoses, 7.2% for ADHD combined presentation, and 0.5% for ADHD inattentive presentation.

*ALSPAC neurodevelopmental outcomes*

After the exclusion of one twin per each pair, the median number of total parent-rated ADHD DAWBA rated traits was 2 (IQR 0-7).

*COPSAC trio analyses*

The maternal indirect genetic effect on ADHD trait score detected within the COPSAC cohort was attenuated after adjustment for social circumstances variables and putative predisposing factors for ADHD. When restricting the adjustment to variables significantly associated with the maternal dietary pattern PGS (maternal and paternal educational level) the estimate remained significant however with a confidence interval close to including an effect size of zero: beta = -0.92; 95%CI = -1.84 – -0.00, *p* = 0.050. Although smoking was significantly associated with the maternal dietary pattern PGS, we did not include this as a covariate in the model, since existing literature suggests the observed association between maternal smoking in pregnancy and offspring ADHD to be spurious and a product of genetic confounding.^3^

*MoBa sensitivity analyses*

In the larger MoBa cohort main trio analyses were performed using robust standard errors clustered on maternal ID. This did not alter the results. Changing the diagnostic grouping of ADHD diagnoses from requiring only one to two registered diagnoses in the Norwegian Patient Registry did not alter the results either.

# Supplementary tables and figures

**Supplementary Figure 1.** Flow chart of the COPSAC_2010_ study cohort

COPSAC2010 Cohort

N = 700

Completed COPSYCH evaluation

N = 604

Exclusions:

Twin B

Non-biological parents

Missing data

N remaining:

Maternal PGS: 528

Paternal PGS: 449

Child PGS: 528

Information on research diagnosis of ADHD

N = 593

Complete Trios

N = 437

Information on PGS for at least 1 family member

N = 530

| **Supplementary Table 1: Drop out analysis** | | | | |
| --- | --- | --- | --- | --- |
|  | Total cohort | Incomplete Trios | Complete trios | P-value* |
|  | n =700 | n =263 | n = 437 |  |
| Child dietary pattern PGS, mean (SD) | 0.04 (1.00) | 0.02 (0.95) | 0.05 (1.03) | 0.531 |
| Maternal dietary pattern PGS, mean (SD) | -0.04 (1.03) | 0.08 (1.01) | -0.08 (1.03) | 0.078 |
| Paternal dietary pattern PGS, mean (SD) | 0.05 (0.98) | 0.12 (0.97) | 0.03 (0.99) | 0.299 |
| Sex, male, n (%) | 360 (51.4) | 139 (52.9) | 221 (50.6) | 0.426 |
| Gestational age, d, mean (SD) | 279.00 (11.65) | 278.46 (12.81) | 279.33 (10.89) | 0.233 |
| Birthweight, kg, mean (SD) | 3.54 (0.55) | 3.51 (0.59) | 3.55 (0.52) | 0.271 |
| Maternal educational level, n (%) |  |  |  | 0.449 |
| Elementary school | 20 (2.9) | 6 (2.3) | 14 (3.2) |  |
| College graduate | 35 (5.0) | 11 (4.2) | 24 (5.5) |  |
| Tradesman certification | 130 (18.6) | 45 (17.1) | 85 (19.5) |  |
| Bachelor’s' degree | 321 (45.9) | 129 (49.0) | 192 (43.9) |  |
| Masters' degree or higher | 194 (27.7) | 72 (27.4) | 122 (27.9) |  |
| Paternal educational level, n (%) |  |  |  | 0.128 |
| Elementary school | 31 (4.6) | 14 (5.7) | 17 (3.9) |  |
| College graduate | 39 (5.7) | 13 (5.3) | 26 (6.0) |  |
| Tradesman certification | 213 (31.3) | 64 (26.2) | 149 (34.2) |  |
| Bachelor’s' degree | 206 (30.3) | 83 (34.0) | 123 (28.2) |  |
| Masters' degree or higher | 191 (28.1) | 70 (28.7) | 121 (27.8) |  |
| Household income, n (%) |  |  |  | 0.129 |
| <100.000 DKK | 67 (9.6) | 34 (13.0) | 33 (7.6) |  |
| 100.000-150.000 DKK | 170 (24.3) | 63 (24.0) | 107 (24.5) |  |
| 150.000-200.000 DKK | 200 (28.6) | 68 (26.0) | 132 (30.2) |  |
| 200.000-250.000 DKK | 157 (22.5) | 57 (21.8) | 100 (22.9) |  |
| >250.000 DKK | 105 (15.0) | 40 (15.3) | 65 (14.9) |  |
| Alcohol intake in pregnancy, yes, n (%) | 101 (14.5) | 35 (13.4) | 66 (15.1) | 0.411 |
| Smoking in pregnancy, yes, n (%) | 54 (7.7) | 24 (9.1) | 30 (6.9) | 0.242 |
| Parity, n (%) |  |  |  | 0.071 |
| 1 | 323 (46.1) | 132 (50.2) | 191 (43.7) |  |
| 2 | 267 (38.1) | 86 (32.7) | 181 (41.4) |  |
| ≥3 | 110 (15.7) | 45 (17.1) | 65 (14.9) |  |
| Maternal pre-pregnancy BMI, mean (SD) | 24.55 (4.39) | 24.19 (4.18) | 24.76 (4.50) | 0.070 |
| Maternal Western dietary pattern PC, mean (SD) | -0.1 (0.99) | -0.12 (1.05) | 0.05 (0.96) | 0.046 |
| Maternal age, y, mean (SD) | 32.28 (4.36) | 32.67 (4.62) | 32.05 (4.18) | 0.069 |
| Paternal age, y, mean (SD) | 34.51 (5.27) | 34.48 (5.52) | 34.53 (5.13) | 0.629 |
| Vitamin D intervention, yes, n (%) | 297 (50.9) | 122 (55.5) | 175 (48.1) | 0.069 |
| Fish oil intervention, yes, n (%) | 347 (49.7) | 114 (43.5) | 233 (53.4) | 0.014 |
| *The p-value tests for differences between incomplete and complete trios | | | | |

**Supplementary Figure 2.** Top 20 food groups associated with the maternal dietary pattern PGS and their association with COPSAC maternal Western dietary pattern PC. The maternal Western dietary pattern PC and its association to pregnancy dietary intake has been presented previously by Horner et al and associated to neurodevelopmental conditions.^4^

**Supplementary Figure 3.** Top 20 food groups associated with the COPSAC maternal Western dietary pattern PC and their association with maternal dietary pattern PGS. The maternal Western dietary pattern PC and its association to pregnancy dietary intake has been presented previously by Horner et al and associated to neurodevelopmental conditions.^4^

**Supplementary Figure 4.** Mean COPSAC maternal Western dietary pattern PC plotted against intervals of the maternal dietary pattern PGS. Mothers with higher values of the COPSAC Western dietary pattern PC, previously associated with offspring neurodevelopmental disorders by Horner et al., displayed lower values of the dietary pattern PGS i.e. the genetic disposition to a healthy dietary pattern.

| **Supplementary Table 2: Association between selected social circumstance and**  **prenatal exposure variables** **and maternal dietary pattern PGS** | | | |
| --- | --- | --- | --- |
|  | n | β [95%CI] | p-value |
| Sex, male | 597 | -0.04 [-0.20;0.13] | 0.657 |
| Child birth weight, kg | 597 | 0.04 [-0.11;0.20] | 0.610 |
| Child gestational age, days | 597 | 0.00 [-0.01;0.00] | 0.382 |
| Maternal smoking in pregnancy | 597 | -0.41 [-0.73;-0.10] | 0.010 |
| Maternal alcohol intake in pregnancy | 595 | 0.00 [-0.23;0.24] | 0.982 |
| Maternal parity, n (1, 2, ≥3) | 597 | 0.03 [-0.09;0.14] | 0.608 |
| Maternal use of antibiotics in pregnancy | 596 | 0.06 [-0.11;0.23] | 0.481 |
| Maternal pre-eclampsia in pregnancy | 596 | 0.01 [-0.39;0.41] | 0.960 |
| Paternal age at birth, years | 583 | 0.02 [-0.00;0.03] | 0.064 |
| Maternal CRP at pregnancy week 24, log2 transformed | 594 | -0.03 [-0.09;0.02] | 0,228 |
| Maternal glycA at pregnancy week 24 | 595 | -0.61 [-1.43;0.20] | 0,137 |
| Maternal pre-pregnancy BMI | 594 | 0.00 [-0.02;0.02] | 0.999 |
| Maternal age at child birth, years | 597 | 0.01 [-0.01;0.03] | 0.220 |
| Maternal education at child birth | 597 |  | 0.002* |
| Elementary |  | Ref |  |
| College |  | 0.18 [-0.41;0.77] |  |
| Tradesman |  | 0.08 [-0.42;0.57] |  |
| Medium |  | 0.25 [-0.22;0.73] |  |
| University |  | 0.54 [0.06;1.02] |  |
| Paternal education at child birth | 582 |  | <0.001* |
| Elementary |  | Ref |  |
| College |  | 0.81 [0.29;1.33] |  |
| Tradesman |  | 0.36 [-0.07;0.78] |  |
| Medium |  | 0.53 [0.11;0.96] |  |
| University |  | 0.69 [0.27;1.12] |  |
| Household income at child birth | 596 |  | 0.312* |
| <100.000 |  | -0.08 [-0.41;0.25] |  |
| 100.000-150.000 |  | Ref |  |
| 150.000-200.000 |  | 0.11 [-0.11;0.34] |  |
| 200.000-250.000 |  | 0.13 [-0.11;0.37] |  |
| >250.000 |  | 0.25 [-0.03;0.52] |  |
| Linear regression model used to test association between continuous covariates and dietary pattern polygenic score.  BMI = Body Mass Index. CRP = C-reactive protein. *P-value from likehood ratio test | | | |

**Supplementary Figure 5.** Maternal dietary pattern polygenic score (PGS) plotted against maternal diet as reported by food frequency questionnaires in the MoBa cohort. The plot depicts the 20 most positively and the 20 most negatively associated food groups. FDR significant p-values are marked with an asterisk (*).

**Supplementary Figure 6.** Maternal dietary pattern polygenic score (PGS) plotted against maternal diet as reported by food frequency questionnaires in the ALSPAC cohort. Only nominal significant associations were included in the figure. None of the associations were significant after FDR-correction.

| **Supplementary Table 3: Dietary pattern PC1 derived from separate PC analyses**  **of the three included birth cohorts COPSAC, MoBa and ALSPAC** | | |
| --- | --- | --- |
| Birth Cohort | Dietary pattern PC1,  Explained variation in pregnancy dietary intake | Correlation between  maternal dietary pattern PGS and PC1 |
| **COPSAC** | 8.6% | R = 0.31, R^2^ = 0.097, p = < 0.001 |
| **MoBa** | 5.1% | R = -0.18, R^2^ = 0.032, p < 0.001 |
| **ALSPAC** | 1.0% | R = -0.02, R^2^ = 0.001, p = 0.053 |
| Numbers included in analyses: COPSAC n = 515, MoBa n = 72,016, ALSPAC n = 7,006. | | |

**Supplementary Figure 7:** COPSAC_2010_ plot of the first and second principal component

**Supplementary Figure 8:** MoBa plot of the first and second principal component

**Supplementary Figure 9:** ALSPAC plot of the first and second principal component

| **Supplementary Table 4: Association between maternal, paternal, and child dietary pattern PGS and offspring ADHD diagnosis and ADHD-RS traits in the COPSAC**_2010_ **cohort** | | | | | | | |
| --- | --- | --- | --- | --- | --- | --- | --- |
|  | **Maternal PGS**  n = 511 | | **Paternal PGS**  n = 449 | | **Child PGS**  n = 528 | | |
|  | **OR [95%CI]** | **p-value** | **OR [95%CI]** | **p-value** | | **OR [95%CI]** | **p-value** |
| **ADHD** | 0.71 [0.53;0.93] | 0.015 | 0.77 [0.57;1.03] | 0.085 | | 0.55 [0.41;0.73] | <0.001 |
| **ADHD**  **Inattentive presentation** | 0.76 [0.51;1.12] | 0.174 | 0.77 [0.51;1.16] | 0.206 | | 0.56 [0.37;0.82] | 0.004 |
| **ADHD**  **Combined presentation** | 0.71 [0.49;1.00] | 0.055 | 0.81 [0.55;1.19] | 0.279 | | 0.60 [0.41;0.86] | 0.006 |
|  | **Maternal PGS**  n = 513 | | **Paternal PGS**  n = 450 | | | **Child PGS**  n = 530 | |
|  | **β [95%CI]** | **p-value** | **β [95%CI]** | **p-value** | | **β [95%CI]** | **p-value** |
| **ADHD-RS**  **Total traits** | -1.23  [-1.93;-0.53] | <0.001 | -0.50  [-1.30;0.29] | 0.212 | | -1.10  [-1.82;-0.38] | 0.003 |
| **ADHD-RS**  **Inattentive traits** | -0.54  [-0.98;-0.11] | 0.015 | -0.25  [-0.74;0.24] | 0.313 | | -0.65  [-1.10;-0.21] | 0.004 |
| **ADHD-RS**  **Impulsivity/Hyperactivity traits** | -0.69  [-1.03;-0.35] | <0.001 | -0.25  [-0.64;0.13] | 0.196 | | -0.45  [-0.80;-0.10] | 0.012 |
| All analyses were adjusted for child sex  ADHD: Attention deficit hyperactivity disorder. ADHD-RS: ADHD Rating Scale. CI: Confidence Interval. OR: Odds ratio. PGS: polygenic score. | | | | | | | |

| **Supplementary Table 5: Trio models of the association between maternal, paternal, and child dietary pattern PGS and offspring ADHD diagnosis and ADHD-RS traits in the COPSAC**_2010_ **cohort** | | | | | | | |
| --- | --- | --- | --- | --- | --- | --- | --- |
|  | **Maternal PGS**  **Trio model***  n = 437 | | **Paternal PGS**  **Trio model****  n = 437 | | **Child PGS**  **Trio model*****  n = 437 | | |
|  | **OR [95%CI]** | **p-value** | **OR [95%CI]** | **p-value** | | **OR [95%CI]** | **p-value** |
| **ADHD** | 0.98  [0.69;1.38] | 0.893 | 1.05 [0.73;1.53] | 0.780 | | 0.58 [0.38;0.88] | 0.011 |
| **ADHD**  **Inattentive presentation** | 1.1  [0.67;1.78] | 0.702 | 1.06 [0.63;1.78] | 0.825 | | 0.57 [0.31;1.02] | 0.063 |
| **ADHD**  **Combined presentation** | 0.89  [0.56;1.39] | 0.603 | 1.03 [0.64;1.67] | 0.893 | | 0.65 [0.37;1.10] | 0.111 |
|  | **Maternal PGS**  **Trio model***  n = 438 | | **Paternal PGS**  **Trio model****  n = 438 | | | **Child PGS**  **Trio model*****  n = 438 | |
|  | **β [95%CI]** | **p-value** | **β [95%CI]** | **p-value** | | **β [95%CI]** | **p-value** |
| **ADHD-RS**  **Total traits** | -1.15  [-2.06;-0.23] | 0.015 | -0.34  [-1.35;0.68] | 0.514 | | -0.14  [-1.25;0.98] | 0.810 |
| **ADHD**  **Inattentive traits** | -0.35  [-0.92;0.23] | 0.235 | 0.00  [-0.63;0.63] | 0.995 | | -0.33  [-1.03;0.36] | 0.345 |
| **ADHD**  **Impulsivity/Hyperactivity traits** | -0.80  [-1.24;-0.36] | <0.001 | -0.34  [-0.83;0.15] | 0.171 | | 0.20  [-0.34;0.74] | 0.467 |
| *Adjusted for child sex and child PGS  **Adjusted for sex, maternal PGS and child PGS  ***Adjusted for sex, maternal PGS and paternal PGS  ADHD: Attention deficit hyperactivity disorder. ADHD-RS: ADHD Rating Scale. CI: Confidence Interval. OR: Odds ratio. PGS: polygenic score. | | | | | | | |

**Supplementary Figure 10.** Unadjusted and trio models of the dietary pattern PGS association to ADHD-RS traits after exclusion of individuals with a diagnosis of ADHD in the COPSAC_2010_ cohort

**Supplementary Figure 11.** Unadjusted and trio models of the dietary pattern PGS association to ADHD diagnosis and ADHD-RS traits in the COPSAC_2010_ cohort including additional adjustment for child, maternal, and paternal ADHD PGS

**Supplementary Figure 12.** Unadjusted and trio models of the association between dietary pattern PGS and NPR ADHD diagnosis as well as ADHD RS-DBD traits in the MoBa cohort

**Supplementary Figure 13.** Unadjusted and trio models of the association between dietary pattern PGS and DAWBA ADHD traits in the ALSPAC cohort

| **Supplementary Table 6: Association between maternal, paternal, and child dietary pattern PGS and offspring ADHD NPR diagnosis and RS-DBD ADHD traits in the MoBa cohort** | | | | | | | |
| --- | --- | --- | --- | --- | --- | --- | --- |
|  | **Maternal PGS**  n = 91,931 | | **Paternal PGS**  n = 62,353 | | **Child PGS**  n = 75,342 | | |
|  | **OR [95%CI]** | **p-value** | **OR [95%CI]** | **p-value** | | **OR [95%CI]** | **p-value** |
| **ADHD** | 0.95 [0.92;0.97] | <0.001 | 0.95 [0.92;0.98] | <0.001 | | 0.92 [0.89;0.94] | <0.001 |
| **ADHD**  **Inattentive presentation** | 0.97 [0.88;1.06] | 0.485 | 0.95 [0.85;1.07] | 0.401 | | 1.00 [0.90;1.11] | 0.933 |
| **ADHD**  **Combined presentation** | 0.94 [0.92;0.97] | <0.001 | 0.95 [0.92;0.98] | 0.001 | | 0.91 [0.89;0.94] | <0.001 |
|  | **Maternal PGS**  n = 36,131 | | **Paternal PGS**  n = 26,619 | | | **Child PGS**  n = 31,191 | |
|  | **β [95%CI]** | **p-value** | **β [95%CI]** | **p-value** | | **β [95%CI]** | **p-value** |
| **RS-DBD**  **Total traits** | -0.08  [-0.15;-0.01] | 0.031 | -0.13  [-0.21;-0.04] | 0.003 | | -0.25  [-0.33;-0.17] | <0.001 |
| **RS-DBD**  **Inattentive traits** | -0.03  [-0.98;-0.11] | 0.173 | -0.05  [-0.10;-0.01] | 0.026 | | -0.12  [-0.17;-0.08] | <0.001 |
| **RS-DBD**  **Impulsivity/Hyperactivity traits** | -0.05  [-0.09;-0.01] | 0.009 | -0.07  [-0.11;-0.02] | 0.003 | | -0.13  [-0.17;-0.08] | <0.001 |
| All analyses were adjusted for child sex and birth year  ADHD: Attention deficit hyperactivity disorder. CI: Confidence Interval. NPR: Norwegian patient registry. OR: Odds ratio. PGS: polygenic score. RS-DBD: Disruptive Behavior Disorders Rating Scale. | | | | | | | |

| **Supplementary Table 7: Trio models of the association between maternal, paternal, and child dietary pattern PGS and offspring ADHD NPR diagnosis and RS-DBD ADHD traits in the MoBa cohort** | | | | | | | |
| --- | --- | --- | --- | --- | --- | --- | --- |
|  | **Maternal PGS**  **Trio model***  n = 41,580 | | **Paternal PGS**  **Trio model****  n = 41,580 | | **Child PGS**  **Trio model*****  n = 41,580 | | |
|  | **OR [95%CI]** | **p-value** | **OR [95%CI]** | **p-value** | | **OR [95%CI]** | **p-value** |
| **ADHD** | 0.99 [0.95;1.04] | 0.784 | 1.01 [0.96;1.05] | 0.790 | | 0.92 [0.87;0.96] | 0.002 |
| **ADHD**  **Inattentive presentation** | 1.01 [0.85;1.20] | 0.894 | 1.00  [0.84;1.194] | 0.975 | | 0.97 [0.79;1.18] | 0.749 |
| **ADHD**  **Combined presentation** | 0.99 [0.94;1.04] | 0.613 | 1.01 [0.96;1.06] | 0.802 | | 0.92 [0.87;0.97] | 0.002 |
|  | **Maternal PGS**  **Trio model***  n = 18,629 | | **Paternal PGS**  **Trio model****  n = 18,629 | | | **Child PGS**  **Trio model*****  n = 18,629 | |
|  | **β [95%CI]** | **p-value** | **β [95%CI]** | **p-value** | | **β [95%CI]** | **p-value** |
| **RS-DBD**  **Total traits** | 0.09  [-0.03;0.21] | 0.156 | -0.03  [-0.15;0.10] | 0.686 | | -0.28  [-0.42;-0.14] | <0.001 |
| **RS-DBD**  **Inattentive traits** | 0.06  [-0.01;0.13] | 0.117 | -0.01  [-0.08;0.06] | 0.843 | | -0.15  [-0.20;-0.05] | 0.002 |
| **RS-DBD**  **Impulsivity/Hyperactivity traits** | 0.03  [-0.04;-0.10] | 0.382 | -0.02  [-0.08;0.05] | 0.613 | | -0.12  [-0.80;-0.10] | 0.012 |
| *Adjusted for sex, birth year, and child PGS  **Adjusted for sex, birth year, maternal PGS, and child PGS  ***Adjusted for sex, birth year, maternal PGS, and paternal PGS  ADHD: Attention deficit hyperactivity disorder. CI: Confidence Interval. NPR: Norwegian patient registry. OR: Odds ratio. PGS: polygenic score. RS-DBD: Disruptive Behavior Disorders Rating Scale. | | | | | | | |

| **Supplementary Table 8: Association between maternal, paternal, and child dietary pattern PGS and offspring DAWBA ADHD traits in the ALSPAC cohort** | | | | | | | |
| --- | --- | --- | --- | --- | --- | --- | --- |
|  | **Maternal PGS**  n = 5,587 | | **Paternal PGS**  n = 1,517 | | **Child PGS**  n = 6,018 | | |
|  | **β [95%CI]** | **p-value** | **β [95%CI]** | **p-value** | | **β [95%CI]** | **p-value** |
| **DAWBA**  **Total traits** | -0.03  [-0.16;0.10] | 0.650 | -0.20  [-0.43;0.04] | 0.099 | | -0.16  [-0.29;-0.04] | 0.010 |
| **DAWBA**  **Inattentive traits** | -0.01  [-0.10;0.09] | 0.910 | -0.08  [-0.25;0.09] | 0.333 | | -0.11  [-0.20;-0.01] | 0.023 |
| **DAWBA Impulsivity/Hyperactivity traits** | -0.06  [-0.15;0.03] | 0.210 | -0.16  [-0.32;0.01] | 0.060 | | -0.13  [-0.22;-0.04] | 0.004 |
| All analyses were adjusted for child sex  ADHD: Attention deficit hyperactivity disorder. CI: Confidence Interval. DAWBA: Development and Well-Being Assessment. PGS: polygenic score. | | | | | | | |

| **Supplementary Table 9: Trio models of the association between maternal, paternal, and child dietary pattern PGS and offspring DAWBA ADHD traits in the ALSPAC cohort** | | | | | | | |
| --- | --- | --- | --- | --- | --- | --- | --- |
|  | **Maternal PGS**  **Trio model***  n = 1,211 | | **Paternal PGS**  **Trio model****  n = 1,211 | | **Child PGS**  **Trio model*****  n = 1,211 | | |
|  | **β [95%CI]** | **p-value** | **β [95%CI]** | **p-value** | | **β [95%CI]** | **p-value** |
| **DAWBA**  **Total traits** | -0.01  [-0.33;-0.32] | 0.974 | -0.15  [-0.47;0.17] | 0.362 | | -0.10  [-0.47;-0.28] | 0.612 |
| **DAWBA**  **Inattentive traits** | 0.12  [-0.11;0.35] | 0.316 | 0.01  [-0.24;0.22] | 0.949 | | -0.13  [-0.40;0.14] | 0.347 |
| **DAWBA Impulsivity/Hyperactivity traits** | -0.03  [-0.26;0.19] | 0.777 | -0.10  [-0.32;0.12] | 0.382 | | -0.10  [-0.36;0.16] | 0.438 |
| *Adjusted for child sex and child PGS  **Adjusted for sex, maternal PGS and child PGS  ***Adjusted for sex, maternal PGS and paternal PGS  ADHD: Attention deficit hyperactivity disorder. CI: Confidence Interval. DAWBA: Development and Well-Being Assessment. PGS: polygenic score. | | | | | | | |

# Supplementary References

1.	Ge, T., Chen, C.-Y., Ni, Y., Feng, Y.-C. A. & Smoller, J. W. Polygenic prediction via Bayesian regression and continuous shrinkage priors. *Nat. Commun.* **10**, 1776 (2019).

2.	Emmett, P. M., Jones, L. R. & Golding, J. Pregnancy diet and associated outcomes in the Avon Longitudinal Study of Parents and Children. *Nutr. Rev.* **73**, 154–174 (2015).

3.	Faraone, S. V. *et al.* The World Federation of ADHD International Consensus Statement: 208 Evidence-based conclusions about the disorder. *Neurosci. Biobehav. Rev.* **128**, 789–818 (2021).

4.	Horner, D. *et al.* A western dietary pattern during pregnancy is associated with neurodevelopmental disorders in childhood and adolescence. *Nat. Metab.* https://doi.org/10.1038/s42255-025-01230-z (2025) doi:10.1038/s42255-025-01230-z.
